# Impact of Intermittent Fasting and/or Caloric Restriction on Aging-Related Outcomes in Adults: A Scoping Review of Randomized Controlled Trials

**DOI:** 10.3390/nu16020316

**Published:** 2024-01-20

**Authors:** Dara L. James, Nanako A. Hawley, Alex E. Mohr, Janice Hermer, Edward Ofori, Fang Yu, Dorothy D. Sears

**Affiliations:** 1 Edson College of Nursing and Healthcare Innovation, Arizona State University, Phoenix, AZ 85004, USA; fang.yu.2@asu.edu; 2 Department of Psychology, College of Arts and Sciences, University of South Alabama, Mobile, AL 36688, USA; nah2021@jagmail.southalabama.edu; 3 College of Health Solutions, Arizona State University, Phoenix, AZ 85004, USA; aemohr@asu.edu (A.E.M.); edward.ofori@asu.edu (E.O.); dorothy.sears@asu.edu (D.D.S.); 4 Arizona State University Library, Arizona State University, Phoenix, AZ 85004, USA; janice.hermer@asu.edu

**Keywords:** dietary fasting, energy restriction, circadian rhythm, time-restricted eating, nightly fasting

## Abstract

Intermittent fasting (IF) and caloric restriction (CR) are dietary strategies to prevent and attenuate obesity associated with conditions and aging-related outcomes. This scoping review examined the cardiometabolic, cancer, and neurocognitive outcome differences between IF and CR interventions among adults. We applied a systematic approach to scope published randomized controlled trials (databases: PubMed, CINAHL Plus, PsychInfo, Scopus, and Google Scholar) from inception through August 2023. The initial search provided 389 unique articles which were critically appraised. Thirty articles met the eligibility criteria for inclusion: 12 were IF, 10 were CR, and 8 were combined IF and CR interventions. IF and CR were associated with weight loss; however, IF studies tended to report greater adherence compared with CR. Overall, IF and CR were equivalently effective across cardiometabolic, cancer, and neurocognitive outcomes. Our findings suggest that IF has health benefits in a variety of conditions and may be better accepted and tolerated than CR, but more comparative research is required.

## 1. Introduction

Dietary fasting and energy deprivation have been ever-present evolutionary and historical experiences among human populations. Modern society has afforded food surplus and the potential for overconsumption, both associated with the rising prevalence of obesity and aging-related chronic diseases. Humans have evolved to undergo periods of food scarcity and involuntary fasting (i.e., periods of time without food) [1,2]. Food restriction and voluntary fasting have been widely practiced across many cultures throughout history for religious, medicinal, and traditional purposes [3,4]. Within the context of modern Western society, adapted practices of these recurrent human experiences have been formed, chiefly under the practice of intermittent fasting (IF) and/or calorie restriction (CR) [4,5].

While there are instances of irregular food availability and food insecurity, these are considered involuntary circumstances and do not necessarily involve caloric restriction [6]. Intermittent fasting, centered on eating time and frequency, involves voluntary abstinence of caloric consumption over specific periods of hours and/or days and does not necessarily involve calorie restriction [7,8]. CR entails an overall reduction in daily caloric intake, generally >20% less than a normative energy intake, and does not necessitate intake to occur during any specific time domain for energy intake [5,9,10,11]. While CR has long held a prominent standing in the fields of longevity and obesity prevention [5], CR at the recommended reduction in energy intake is likely not sustainable in the long term for most humans. IF has emerged as a viable and rapidly moving field recognized as a dietary strategy and potential alternative to CR [12]. Therefore, the more recent exploration of IF in humans warrants significant transdisciplinary attention and evaluation in comparison with CR for similarities and differences in health and longevity domains. Such evaluations are particularly relevant as more randomized control trials (RCTs) are available to collate for systematic synthesis.

The conceptual framework and mechanistic rationale of IF regimens differ substantially from CR. IF establishes a predetermined timeframe of caloric consumption rather than counting or tracking calorie intake. While IF models can include a secondary emphasis on calories or macronutrients, it is not a requirement [7]; rather, it is the eating behavior (i.e., the timing of eating and fasting) that is paramount. While not appropriate for all populations (e.g., individuals with active/history of eating disorders, frailty, pregnancy, or advanced age), both IF and CR strategies are generally well-tolerated and demonstrate acceptable safety profiles [13,14]. However, IF may afford increased adherence and long-term sustainability [15].

IF is an umbrella term that includes many different regimens. Three general examples are (1) Prolonged nightly fasting (PNF), which promotes food intake during a specific interval of time that is in alignment with biological cycles of circadian rhythm, i.e., calorie consumption during waking hours and abstinence during the nighttime; (2) Alternate day fasting (ADF), which supports ad libitum energy intake on alternating days coupled with fasting days, i.e., no caloric consumption; and (3) Time-Restricted Eating (TRE), which dictates windows of specific time lengths allotted each day for eating and fasting (see Table 1 Review of terminology). An individual typically has flexibility in selecting eating timeframes in a TRE protocol as long as the eating window is restricted and consistent. Other IF protocols have been more recently termed ‘periodic fasting’ (PF) and may involve fasting for several days (e.g., two to seven days) repeated once per month or heavy restriction of a specific macronutrient (i.e., protein) [8]. Collectively, these IF regimens have been implemented in RCTs that form a growing body of research suggesting that IF supports the modulation of favorable shifts in health outcomes.

Both IF and CR have been reported as geroprotective and have been employed to buffer against cardiometabolic perturbation, cancer, neurocognitive decline, and various other ailments associated with obesity and aging and may promote life extension [5,8]. For example, these practices may better maintain blood glucose and lipid metabolism [16,17], induce neurotrophic and autophagic responses [18,19], and increase the production of important metabolites (e.g., ketones and brain-derived neurotrophic factor) [20,21] that may promote reductions in oxidative stress and inflammation [22]. Such changes, over time, are expected to improve cardiometabolic status, cancer, and neurocognition.

As the number of RCTs investigating IF and CR has recently increased, the current review sought to contextualize the current body of literature better, combining outcomes into aging-related domains and mapping key comparisons for IF and CR. We implemented a scoping review approach with the aim of investigating geroprotective domains of cardiometabolic, cancer, and neurocognitive outcomes. As a guide for study element mapping and data collation, our main questions were:

➢ What aging-related outcomes have been examined in RCTs of IF and CR?
➢ What are the within-study effects of IF and CR on cardiometabolic, cancer-specific, and neurocognitive outcomes compared with controls in adults?
➢ What are the differences in the effects of CR versus IF RCT interventions on neurocognitive, cardiometabolic, and cancer domain-specific outcomes in adults?

The limitations of this broad approach notwithstanding, utilizing a systematic process for both IF and CR provides a high-level perspective and encompassing grasp on the current state of the human RCT literature. Such efforts are important in moving beyond narrative synthesis and establishing a more formalized framework for future systematic methods (i.e., meta-analyses) and future RCT construction.

## 2. Materials and Methods

### 2.1. Protocol and Registration

**Figure 1:**
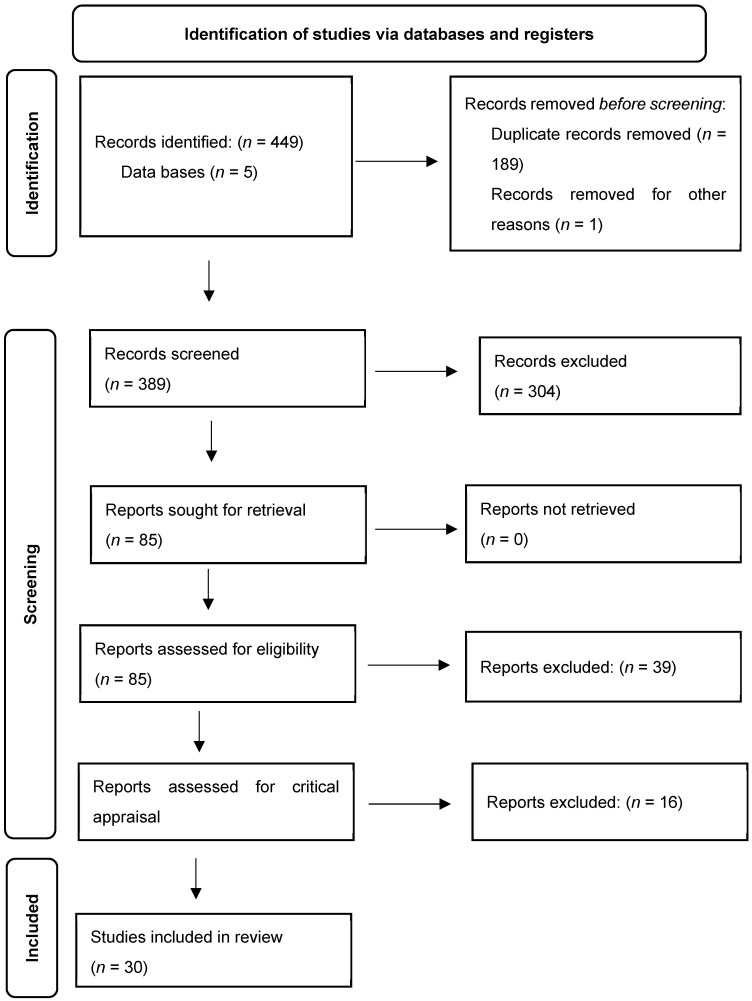
Preferred Reporting Items for Systematic Reviews and Meta-Analyses (PRISMA) flow diagram for the scoping review screening process [25].

This review followed the Preferred Reporting Items for Systematic Reviews and Meta-Analyses (PRISMA) extension for scoping review guidelines [23] (see Figure 1). and the recommendations of the Cochrane Collaboration in the preparation of this scoping review [24]. All methods used in relation to the research questions, search strategy and process, inclusion/exclusion criteria, and risk of bias assessment have been deposited and registered prior to the literature search on Open Science Framework (https://osf.io/ns8am/, accessed on 25 April 2022).

### 2.2. Data Search

This comprehensive literature search was performed by a librarian on 12 and 13 May of 2022, and was conducted using the following English-language databases: PubMed, Cumulative Index to Nursing and Allied Health Literature (CINAHL) Plus with full text, PsycInfo, Scopus, and Google Scholar. An updated search was also performed on 17–18 August 2023. The search included literature published between journal indexing and was not limited by language; subject headings were substituted as appropriate. Below is listed the search strategy for Scopus; all other searches were based on these keywords:

(TITLE-ABS-KEY ({healthy aging} OR {cognitive aging} OR aging OR “age associated disease” OR “age associated diseases” OR “age related disease” OR “age related diseases”) AND TITLE-ABS-KEY (“intermittent fasting” OR {alternate day fasting} OR {time restricted feeding} OR {time restricted eating} OR {alternate day fasting} OR {prolonged overnight fasting} OR {periodic fasting} OR {metabolic switching} OR omad OR {one meal a day} OR {restricted diet} OR {calorie restriction} OR {caloric restriction} OR “low calorie diet” OR {restricted diet} OR “restrictive diet”) AND TITLE-ABS-KEY (“randomized controlled trial” OR “randomised controlled trial” OR rct) AND NOT TITLE-ABS-KEY (animal* OR rats OR rat OR mouse OR mice OR rodent* OR dogs OR dog OR cat OR cats )). In addition, where appropriate, selected reviews and included RCTs were hand-searched to capture any articles missed during the database searches.

All citations were sent to Zotero version 6.0.30 to check for retracted papers and then saved into Covidence™ (https://www.covidence.org; accessed on 24 June 2022) for future screening. At the full-text screening stage, any references without attached PDFs were searched and attached to Covidence by the librarian. Each citation was blind screened by two reviewers at each screening level. Conflicts were decided by a third reviewer.

### 2.3. Eligibility Criteria

Briefly, the population of interest was human adults. The interventions included in the current scoping review were any form of IF, CR, or a combination and/or comparison of both. Control groups were required to continue their regular eating and exercise habits. Finally, the outcomes of interest included cardiometabolic, cancer, and neurocognitive factors. Articles were required to be RCTs.

### 2.4. Screening and Data Extraction

Abstracts found during the search process were exported to Covidence, a web-based platform for managing systematic reviews, and screened for eligibility [D.L.J., A.E.M., and J.H.]; potentially eligible articles were read in full text and examined independently to determine if a given study met the predetermined criteria [D.L.J., N.A.H., A.E.M., and J.H.]. Any disagreements were discussed with the intent to resolve the issue(s) and reach a consensus; as needed, an additional author [D.D.S.] was consulted for further discussion and final decision.

Data were independently extracted [D.L.J., N.A.H., A.E.M., and J.H.] using a data table developed a priori. Data extracted included subject characteristics, study duration, intervention, study design, and outcomes. In line with the full-text selection process, authors [D.L.J., N.A.H., A.E.M., and J.H.] double-checked that these extracted data were correct, and further, any disagreements were discussed with the intent to resolve the issue(s) and reach consensus.

## 3. Results

### 3.1. Search Results

A total of 30 articles were included in the current review, as outlined in the search and screening process in Figure 1. Briefly, the initial search provided 449 records from PubMed, CINAHL Plus with full text, PsycInfo, Scopus, and Google Scholar databases; the updated search provided 129 records. After removing duplicate records from both searches (*n* = 189), 389 unique records were then screened in Covidence by title and abstract. Based on the defined inclusion criteria, 304 records were excluded. The remaining 85 articles were then full-text screened, excluding 39, leaving 46 for critical appraisal. Finally, these articles were evaluated by critical appraisal, ultimately leaving a total of 30 articles for inclusion in the current scoping review.

### 3.2. Study Characteristics

Individual study characteristics, including the key data elements of the intervention, study design and duration, participant population, and outcomes, are outlined below according to IF, CR, and IF and CR combined and/or compared regimens. Respectively, of the 30 included studies, 27 used a parallel group design, and 3 used a crossover design. These studies were conducted in the United States [26,27,28,29,30,31,32,33,34,35,36,37,38,39,40,41,42], Germany [43,44], Malaysia [45,46,47], China [48,49], the United Kingdom [50], Norway [51], Sweden [52], Poland [53], Korea [54], and Austria [55]. Of the total extracted studies, 10 were CR interventions, 12 were IF interventions, and 8 combined and/or compared both IF and CR. In relation to study length, 1 study was short-term (i.e., ≤4 weeks) [55], 14 medium-term (4–12 weeks) [27,28,31,36,39,45,46,47,48,49,51,52,53,54], and 15 longer-term (>12 weeks) [26,29,30,32,33,34,35,37,38,40,41,42,43,44,50]. Populations assessed included healthy men [45,46], healthy individuals [28,31,39], healthy non-obese individuals [29,33,34,35,55], healthy sedentary individuals [40], obese/overweight individuals [26,30,38,41,42,49,50,51,54], overweight men with prediabetes [36], healthy overweight individuals [27,37], overweight non-smokers [44], non-smoking women over 60 years-old [53], overweight non-diabetic individuals [32], obese postmenopausal women [43], and individuals with non-alcoholic fatty liver disease (NAFLD) [47,48,52]. Adherence for long-term studies lasting over 6 months was generally reported as ranging from 5.26% to 21.28% for CR intervention groups and 0–11.54% for control groups in CR studies [29,33,34,37,40]. For IF studies, adherence was 23.8% to 24% for IF groups and 4.76% to 37.7% for control groups [26,38,41,44,50]. For studies comparing multiple interventions, CR groups had an adherence of 15.1% to 17.4%, IF groups 8.88% to 26.47%, control groups 3.85% to 19.35%, and 55% when interventions were combined [38,44].

### 3.3. CR Interventions

Overall, 10 of the included studies investigated CR with the use of a parallel group design (Table 2).

**Table 2:** Characteristics of included studies that implemented calorie restriction interventions.

Reference	Participant Characteristics ^a^	Intervention(s)	Duration	Design	Outcome(s) ^b^
~Calorie Restriction (*n* = 10)~					
Dengo et al., 2010 [28]	Healthy individuals, *n* = 36 (CR, *n* = 25/CON, *n* = 11); 55–75 yrs.; 58% F; BMI: CR, 30.0 ± 0.6 kg/m^2^/CON, 31.8 ± 1.4 kg/m^2^	Hypocaloric diet (1200–1500 kcal), based on the US Department of Agriculture food pyramid guidelines	3 mo	Parallel-group RCT	↓ Body weight ↓ Body fat ↓ Abdominal adiposity ↓ Blood pressure ↓ Β-Stiffness index ↓ Carotid-femoral pulse wave velocity
Fontana et al., 2007 [29]	Healthy, nonobese individuals, *n* = 46 (CR, *n* = 18/HL, *n* = 10); 50–60 yrs.; 63% F; BMI: 23.5–29.9 kg/m^2^	↓ kcal intake 16% first 3 mo.; ↓ kcal intake 20% remaining 9 mo.	12 mo	Parallel-group RCT	↓ Body fat ↓ LDL-cholesterol ↓ Total cholesterol/HDL ratio ↓ HOMA-IR index ↓ CRP
Pierce et al., 2008 [32]	Overweight/obese, non-diabetic individuals, *n* = 40 (CR, *n* = 26/CON, *n* = 14); 21–69 yrs.; CR, 42% F/CON, 36% F; BMI: ≥25–<40 kg/m^2^	↓ kcal intake designed to meet a goal of 10% weight loss/12 weeks (>1200 kcal/day)	4 mo	Parallel-group RCT	↓ Body weight ↓ Total and abdominal body fat ↓ Plasma leptin ↓ Oxidized low-density lipoprotein ↑ Brachial artery flow-mediated dilation
Ravussin et al., 2015 [33]	Healthy, nonobese individuals, *n* = 218 (CR, *n* = 143/CON, *n* = 75); 21–51 yrs.; 69.7% F; BMI: 21.9–28.0 kg/m^2^	↓ kcal intake 25%	24 mo	Parallel-group RCT	CR achieved 11.7 ± 0.7% ↓ kcal intake ↓ 10.4 ± 0.4% weight loss ↓ Triiodothyronine ↓ TNF-α No adverse effects on quality of life
Redman et al., 2018 [34]	Healthy, nonobese individuals *n* = 53 (CR, *n* = 34/CON, *n* = 19); 39.8 ± 6.3 yrs.; 68% F; BMI: 22.0–27.9 kg/m^2^	↓ kcal intake 25%	24 mo	Parallel-group RCT	↓ Body weight ↓ Energy expenditure (sleep) ↓ Triiodothyronine ↓ Urinary F2-isoprostane
Tam et al., 2012 [37]	Healthy, overweight individuals, *n* = 23 (CR, *n* = 12/CON, *n* = 11); CR, 38.4 ± 1.6 yrs./CON, 37.7 ± 2.2 yrs.; 54% F; BMI: 27.8 ± 0.7 kg/m^2^	↓ kcal intake 25%	6 mo	Parallel-group RCT	↓ Body weight ↓ Fat mass ↓ Visceral fat ↑ Insulin sensitivity ↓ Leptin
Wei et al., 2017 [39]	Healthy individuals, *n* = 100 (FMD, *n* = 52/CON, *n* = 48); FMD, 42.2 ± 12.5 years/CON, 43.3 ± 11.7 years; FMD, 62.5% F/CON, 63.5% F; BMI: FMD, 27.8 ± 5.1/CON, 26.6 ± 4.9 kg/m^2^	FMD, low in calories, sugars, and protein but high in unsaturated fats	3 mo	Parallel-group RCT	↓ Body weight ↓ Trunk fat ↓ Total body fat ↓ Blood pressure ↓ IGF-1
Prehn et al., 2017 [43]	Obese, postmenopausal, *n* = 37 (CR, *n* = 19/CON, *n* = 18); CR, 61 ± 4 yrs./CON, 61 ± 6 yrs.; 100% F; BMI: CR, 35.0 ± 3.7 kg/m^2^/CON, 34.7 ± 4.3 kg/m^2^	Wks. 1–8: low-caloric formula diet (800 kcal/day); Wks. 9–12: energy-reduced diet	4 mo	Parallel-group RCT	↓ Body weight ↑ Recognition memory ↑ Gray matter volume in the inferior frontal gyrus and hippocampus ↑ Hippocampal resting-state functional connectivity to parietal areas
Weiss et al., 2006 [40]	Healthy sedentary individuals, *n* = 34 (CR, *n* = 21/CON, *n* = 13); 50–60 yrs.; CR, 61% F/CON, 60% F; BMI: 23.5–29.9 kg/m^2^	↓ kcal intake 16% first 3 mo.; ↓ kcal intake 20% remaining 9 mo.	12 mo	Parallel-group RCT	↑ Insulin sensitivity index ↓ Glucose and insulin AUC ↑ Adiponectin ↓ TNF-α/adiponectin
Coutinho et al., 2018 [51]	Obese individuals, *n* = 35 (IER, *n* = 18/CR, *n* = 17); 39 ± 9 yrs.; 93% F; BMI: 36 ± 4 kg/m^2^	↓ kcal intake 33%	3 mo	Parallel-group RCT	↓ Resting metabolic rate (IER) ↑ Basal and postprandial ghrelin (IER) ↓ Basal active GLP-1 (CR)

Note: Study information is presented as reported. ^a^ Groups were excluded in studies that included interventions outside the scope of this review (i.e., exercise). ^b^ Outcome(s) presented as intervention vs. control. Abbreviations: AUC, the area under the curve; BMI, body mass index; CON, control group; CR, calorie restriction; CRP, C-reactive protein; FMD, fasting-mimicking diet; GLP-1, glucagon-like peptide-1; HDL, high-density lipoprotein; HL, healthy lifestyle guidelines; HOMA-IR, Homeostatic Model Assessment for Insulin Resistance; IER, intermittent energy restriction; IGF-1, Insulin-like growth factor 1; LDL, low-density lipoprotein; RCT, randomized controlled trial; TNF-α, tumor necrosis factor-alpha. ↑ = increased or improved; ↓ = decreased or reduced.

Of the ten included studies, eight reported cardiometabolic findings related to weight or fat loss following CR [28,29,32,33,34,37,39,43], with the majority of studies reporting a reduction in body weight [28,32,33,34,37,39,43], three studies reported a reduction in overall body fat [28,29,32], four studies reported a reduction in adiposity or visceral fat deposits targeted in the abdomen [28,32,37,39], and one study reported a reduction in fat mass [37]. In brief, Weiss et al. (2006) conducted a 12-month CR study examining healthy, non-obese, sedentary individuals (*n* = 18) compared with an exercise training intervention (*n* = 18) and a healthy lifestyle control group (*n* = 10). Study results demonstrated that both the CR and the exercise groups had increased levels of adiponectin, a hormone released by adipocytes that aids with insulin sensitivity [40]. Tam et al. (2012) conducted a 6-month RCT among healthy, overweight individuals comparing a CR intervention (i.e., 25% reduction in energy intake; *n* = 12) to a CR + exercise group (i.e., 12.5% reduction in energy intake +12.5% increase in exercise energy expenditure; *n* = 12) compared with a control group (*n* = 11). Study results for the CR and CR + exercise groups led to reduced circulating levels of leptin, another adipokine secreted exclusively by adipocytes [37].

Of the ten studies included, three studies had primary aims related to circulating concentrations of glucose and insulin [29,37,40]. A 12-month study employing CR among healthy, sedentary individuals found a decrease in glucose levels and insulin area under the curve (AUC) [40]. An increase in insulin sensitivity was reported in CR two studies [37,40]. Fontana et al. (2007) conducted a 1-year RCT in 48 non-obese individuals who were assigned to one of three study groups: (1) 20% CR (*n* = 18); (2) 20% increase in energy expenditure through exercise; and (3) healthy lifestyle guideline control group. Results from this study indicated that both intervention groups yielded a decrease in the HOMA-IR index and levels of LDL-cholesterol and total cholesterol/HDL-cholesterol ratio [29]. Coutinho et al. (2017) conducted a 12-week RCT among 35 individuals with obesity comparing two CR groups: (1) continuous energy restriction (*n* = 18) and (2) intermittent energy restriction (*n* = 17) on outcomes of body composition and weight loss induced compensatory responses. A decrease in resting metabolic rate was found after a 3-month period of CR [51]. In a study including healthy, non-obese individuals, a decrease in C-reactive protein (CRP) was found after a 3-month period of CR [29]. A decrease in blood pressure was found in two studies with healthy individuals following a 3-month period of CR [28,39]. Pierce et al. (2008) conducted a 12-week RCT among 40 non-diabetic men and women (ages 21–69) who were overweight or obese comparing a CR intervention group to an attention control group. An increase in brachial artery flow-mediated dilation was found in the CR intervention group [32]. Dengo et al. (2010) conducted a 4-week RCT among midlife and older adults (*n* = 16) who were overweight or living with obesity to compare a weight loss intervention group to a control group to assess measures of arterial stiffness. Outcomes resulted in decreased β-stiffness index and carotid-femoral pulse wave velocity among the CR (i.e., weight loss) group [28].

Only one study that employed a CR regimen investigated cancer outcomes using a fasting-mimicking diet (FMD) (i.e., low in calories, sugar, and protein; high in unsaturated fats) in 100 generally healthy individuals [39]. Participants in the intervention group were asked to consume an FMD for 5 consecutive days for a period of 3 months; comparatively, the control group consumed an unrestricted diet. Investigators found that the regimen reduced levels of insulin-like growth factor 1 (IGF-1), a hormone associated with several types of cancer [56,57], following three FMD cycles within a 3-month period.

In relation, only one study investigated neurocognitive-related outcomes. Prehn et al. (2017) conducted an RCT among postmenopausal women with obesity (*n* = 19) comparing CR intervention (i.e., low-calorie diet and negative energy balance) to a control group (i.e., no dietary changes) on the primary outcomes of neurocognition. Study results indicated improved recognition memory, paralleled by functional connectivity to parietal areas through increased gray matter volume in the inferior frontal gyrus and hippocampus, and augmented hippocampal resting state in the CR group compared with the control group [43].

### 3.4. IF Interventions

All 12 of the included studies that investigated IF had primary aims related to cardiometabolic outcomes, with the majority (seven studies) implementing a form of TRE (Table 3) [27,30,31,35,36,49,53].

**Table 3:** Characteristics of included studies that implemented intermittent fasting interventions.

Reference	Participant Characteristics ^a^	Intervention(s)	Duration	Design	Outcome(s) ^b^
~Intermittent Fasting (*n* = 12)~					
Cienfuegos et al., 2020 [27]	Healthy, overweight individuals, *n* = 49 (4 h IF, *n* = 16/6 h IF, *n* = 19/CON, *n* = 14); 4 h IF, 49 ± 2 yrs./6h IF, 46 ± 3 yrs./CON, 45 ± 2 yrs.; 4 h IF, 88% F/6 h IF 95% F/CON 86% F; BMI: 4 h IF, 36 ± 1 kg/m^2^/6 h IF 37 ± 1 kg/m^2^/CON 36 ± 1 kg/m^2^	4 h and 6 h restricted feeding (TRF)	2.5 mo	Parallel-group RCT	↓ Body weight (4 h IF and 6 h IF) ↓ insulin resistance (4 h IF and 6 h IF) ↓ oxidative stress (4 h IF and 6 h IF)
Lowe et al., 2020 [30]	*n* = 116 (IF, *n* = 59/CON, *n* = 57); IF, 46.5 ± 10.5 yrs./CON, 46.1 ± 10.3 yrs.; IF, 40.7% F/CON 38.6% F; BMI: IF, 32.9 ± 4.9 kg/m^2^/CON, 32.6 ± 3.4 kg/m^2^	16:8 time-restricted eating, eat ad libitum from 12:00 PM–8:00 PM; abstain from caloric intake from 8:00 PM–12:00 PM the following day.	4 mo	Parallel-group RCT	↓ Body weight ↓ Appendicular lean mass
Martens et al., 2020 [31]	Healthy individuals, *n* = 22; 55–79 yrs.; BMI: 24.7 ± 0.6 kg/m^2^	Time-restricted feeding, <8 h/day	1.5 mo	Randomized crossover	↓ hunger No influence on lean mass No influence on bone density ↑ Functional capacity improved ↑ Glucose tolerance improved
Stote et al., 2007 [35]	Healthy normal-weight adults, *n* = 15; 40–50 yrs.; 66.66% F; BMI: 23.4 ± 0.529 kg/m^2^	↓ meal frequency diet; 1 meal/day.	2 mo	Randomized crossover	↑ hunger ↓ fat mass ↑ BP ↑ LDL ↑ HDL ↓ Cortisol
Sutton et al., 2018 [36]	Overweight men with prediabetes, *n* = 8; 56 ± 9 yrs.; BMI: 32.2 ± 4.4 kg/m^2^	eTRF schedule (6-h daily eating period, with dinner before 15:00 h).	1.25 mo	Randomized crossover	↑ Insulin sensitivity ↑ β cell responsiveness ↑ BP
Xie et al., 2022 [49]	Overweight individuals, *n* = 82 (eTRF, *n* = 28/mTRF, *n* = 26/CON, *n* = 28); eTRF, 28.7 ± 3.1 yrs./mTRF, 31.1 ± 8.4 yrs./CON, 33.6 ± 11.6 yrs.; eTRF, 85.7% F/mTRF 73.1% F/CON 75% F; BMI: eTRF, 22.7 ± 3.1 kg/m^2^/mTRF, 21.4 ± 2.2 kg/m^2^/CON, 21.5 ± 2.9 kg/m^2^	eTRF, mTRF	1.25 mo	Parallel-group RCT	↑ Insulin sensitivity ↑ fasting glucose (eTRF) ↓ body mass (eTRF) ↓ adiposity (eTRF) ↓ inflammation (eTRF) ↑ gut microbial diversity (eTRF)
Domaszewski et al., 2020 [53]	Non-smoking women, age > 60, *n* = 45 (IF, *n = 25*/CON, *n* = 20); IF, 65 ± 4.0 yrs./CON, 66 ± 4.7 yrs.; 65 ± 5 yrs.; IF, 100% F/CON 100% F; BMI: 72.4 ± 12.6 kg/m^2^	Abstinence from food intake for 16h/day, from 20:00 p.m. to 12:00 a.m. (the next day)	1.5 mo	Parallel-group RCT	↓ Body weight ↓ Fat Mass
Ezpeleta et al., 2023 [42]	Individuals with obesity and NAFLD, *n* = 39 (ADF, *n* = 19/CON, *n* = 20); ADF, 44 ± 16 yrs./CON, 44 ± 12 yrs.; ADF, 80% F/CON 80% F; BMI: ADF, 36 ± 8 kg/m^2^/CON, 37 ± 5 kg/m^2^	ADF	3 mo	Parallel-group RCT	↓ Body weight ↓ Fat mass ↓ BMI ↓Fasting insulin ↓HOMA-IR ↓Fasting glucose
Cho et al., 2019 [54]	Overweight/obese individuals, *n* = 31 (IF, *n* = 9/CON, *n* = 5); IF, 33.5 ± 5 yrs./CON, 42.6 ± 10.6 yrs.; IF 75% F/CON 40% F; BMI: IF, 27.8 ± 3.4 kg/m^2^/CON 25.8 ± 3.4 kg/m^2^	ADF	2 mo	Parallel-group RCT	↓ Body weight ↓ Fasting glucose ↑ Cholesterol metabolisms
Stekovic et al., 2019 [55]	Healthy, non-obese individuals, *n* = 90 (IF, *n* = 30/CON, *n* = 60)	ADF	1 mo	Parallel-group RCT	↓ fat mass ↑ fat-to-lean ratio ↓ LDL ↓ triodothyronine
Holmer et al., 2021 [52]	Individuals with w/non-alcoholic fatty liver disease, *n* = 49 (IF, *n* = 25/CON, *n* = 24); IF, 57 ± 10 yrs./CON, 56 ±9 yrs.; IF, 48% F/CON 71% F; BMI: IF, 32.3 ± 2.7 kg/m^2^/CON, 32.9 ± 5.2 kg/m^2^	5:2 Diet	3 mo	Parallel-group RCT	↓ steatosis ↓ Body weight ↓ LDL levels ↑ liver stiffness
Bartholomew et al., 2021 [26]	Overweight individuals, *n* = 103 (IF, *n* = 50/CR, *n* = 53); IF, 49.3 ± 12.0 yrs./CON, 47.0 ± 9.8 yrs.; IF, 66% F/CON 67.9% F	24 h water-only fast	6.5 mo (4 weeks: 2×/week; 22 weeks: 1/week)	Parallel-group RCT	↓ HOMA-IR Weight NS BDNF NS

Note: Study information is presented as reported. ^a^ Groups were excluded in studies that included interventions outside the scope of this review (i.e., exercise). ^b^ Outcome(s) presented as intervention vs. control. Abbreviations: ADF, alternate day fasting; BDNF, brain-derived neurotrophic factor; BP, blood pressure; BMI, body mass index; CON, control group; IF, Intermittent fasting; eTRF, early time-restricted feeding; HDL, high-density lipoprotein; HOMA-IR, Homeostatic Model Assessment for Insulin Resistance; IF, intermittent fasting; IHTG, intrahepatic triglyceride; LDL, low-density lipoprotein; mTRF, time-restricted feeding; NAFLD, non-fatty liver disease; RCT, randomized controlled trial; TRF, time-restricted feeding. ↑ = increased or improved; ↓ = decreased or reduced.

Of these, three studies implemented ADF [42,54,55], one study implemented 5:2 MFR [52], and one study implemented a 24-h water fast [26]. The effect of IF on cardiometabolic outcomes included decreased body weight/fat [27,30,35,42,49,52,53,54,55], improvements in blood lipids and glucose metabolism [26,27,35,36,42,49,52,54,55], and decreased oxidative stress or inflammation [27,49]. Several studies reported increased hunger [35,36] and decreased triiodothyronine [55].

Of the IF studies, seven included a form of TRE, or time-restricted feeding (TRF). In brief, in an 8-week RCT, Cienfuegos et al. (2020) compared the effects of a 4-h feeding window to a 6-h feeding window to a control group (i.e., no mealtime parameters) on body weight and cardiometabolic risk factors. Post-intervention outcomes demonstrated comparable outcomes across the two TRF regimens with respect to reduced body weight, insulin resistance, and oxidative stress compared with the control group [27]. Lowe et al. (2020) conducted a 12-week RCT with 116 adults who were randomized to either a TRE group which eating ad libitum (from 12:00 p.m. to 8:00 p.m.), abstaining from caloric restriction outside that window, compared with a consistent meal timing group instructed to eat three meals per day. Results demonstrated that the TRE group had a significant decrease in weight and increased lean mass index compared with the control group [30]. Hajek et al. (2021) also implemented a parallel-group design with three arms consisting of the 5:2 diet with and without behavioral support (target reduction of 500–600 kcal/daily) and a control over a 12-month period. Both intervention groups achieved similar weight loss, though they suffered from high levels of attrition, with 56% completing the 5:2 diet with self-help and 45% completing the 5:2 diet only. Among a group of healthy, non-obese midlife and older adults, Martens et al. (2020) conducted a pilot randomized crossover trial where participants were randomly assigned to engage in 6 weeks of TRF (i.e., self-select staring time for 10–11 h, required to maintain same 8-h feeding window each day) or normal feeding [31]. Among the 24 study participants, TRF was evaluated to be highly adherent, safe, and well-tolerated. In a crossover RCT, Stote et al. (2007) reported a reduction in cortisol in participants fasting other than the consumption of one meal per day over a 6-month period (which did not include caloric restriction) compared with a “control diet” which included the same number of calories divided across three daily meals (i.e., breakfast, lunch dinner) [35]. In a controlled feeding clinical trial, Sutton et al. (2018) randomized prediabetic men to an eTRF regimen (6-h feeding window; dinner before 3:00 p.m.) or a control group with a feeding schedule of a 12-h period (selected by the participant) [36]. Results from this study indicate that the eTRF group showed improved insulin sensitivity, blood pressure, oxidative stress, and appetite compared with the control group. In a recent 2022 5-week RCT, Xie et al. examined two different TRE protocols (i.e., early TRF [eTRF]; *n* = 30; and mid-day TRF [mTRF]; *n* = 30) compared with a control group (*n* = 30) among healthy individuals living without obesity [49]. Study results indicate that eTRF was more effective at improving insulin sensitivity and fasting glucose while reducing body mass and adiposity, ameliorating inflammation, and increasing gut microbial diversity as opposed to mTRF and/or the control group. In a 6-week study of 45 women ages 60 and older, Domaszewski et al. (2020) explored a 16:8 TRF intervention group (i.e., abstinence from food intake 16 h/day from 8:00 p.m. to 12:00 a.m. the next day; *n* = 25) compared with a control group (*n* = 20) that was asked to follow an eating plan based on their previous habits [53]. Study results demonstrated body weight in the TRF group decreased by ~4.4 pounds.

Ezpeleta et al. (2023) conducted a 3-month RCT comparing ADF combined with exercise to ADF alone to exercise alone among adults (*n* = 80; ages 23–65; 81% female) living with obesity and non-fatty acid liver disease [42]. Post-intervention results demonstrated that intrahepatic triglyceride, body weight, fat mass, and waist circumference were all significantly reduced in the ADF + exercise combination group compared with the control group. In a 2019 pilot RCT, Cho et al. examined the effects of ADF and exercise on cholesterol among a group of adults (*n* = 112) living with overweight or obesity [54]. Study findings indicate that exercise, with or without ADF, improved cholesterol. A 2019 study by Stekovic et al. demonstrated that 4 weeks of ADF improved general health markers among middle-aged adults while also initiating a reduction in caloric restriction by 37% [55]. Further, ADF improved cardiovascular markers and reduced fat mass—results indicate that ADF may have positive physiological impacts and is safe to participate in as a non-pharmacological intervention.

No IF studies directly assessed cancer outcomes, though Bartholomew et al. (2021) examined neurocognitive outcomes but did not find a significant difference in BDNF or GCPi levels in participants that underwent a 24-h water-only fasting intervention one to two times per week over a 6.5 month period compared with a control group that ate ad libitum [26]. The participants from the included IF studies were overweight or obese, except for a few instances where they were a healthy normal weight [31,35,55].

### 3.5. IF and CR Interventions Combined and Compared

Overall, 8 studies combined and/or compared IF with CR (Table 4) [38,41,44,45,46,47,48,50] and investigated cardiometabolic outcomes, with the exception of one study that validated mood and quality of life questionnaires implemented by Teng et al. (2011).

**Table 4:** Characteristics of included studies that implemented calorie restriction and intermittent fasting interventions.

Reference	Participant Characteristics ^a^	Intervention(s)	Duration	Design	Outcome(s) ^b^
~Combined Calorie Restriction and Intermittent Fasting (*n* = 8)~					
Trepanowski et al., 2017 [38]	Overweight/obese individuals, *n* = 79 (ADF, *n* = 22/CR, *n*= 29/CON, *n* = 25); ADF, 46 ± 2 yrs./CR, 44 ± 2 yrs./CON, 44 ± 2 yrs.; ADF, 88% F/CR, 79%F/CON, 84%F; BMI: ADF, 34 ± 1. kg/m^2^/CR, 35 ± 1 kg/m^2^/CON, 34 ± 1 kg/m^2^	↓ kcal intake 25%; ADF, 25% of energy needs on fast days, 125% of energy needs on alternating “feast days”	12 mo	Parallel-group RCT	↑ FFM: total mass ratio (ADF and CR) ↓Circulating leptin (ADF and CR)
Lin et al., 2023 [41]	Individuals with obesity, *n* = 77 (TRE, *n* = 26/CR, *n* = 25/CON, *n* = 26); TRE, 44 ± 12 yrs./CR, 44 ± 9 yrs./CON 44 ± 13 yrs.; TRE, 25% F/CR, 24% F/CON 25% F; BMI: TRE, 37 ± 6 kg/m^2^/CR, 37 ± 5 kg/m^2^/CON, 38 ± 5 kg/m^2^	TRE, eating between noon and 8:00 p.m. only, CR, daily energy deficit ↓ 25%	12 mo	Parallel-group RCT	↓ Fat mass (TRE and CR) ↓ Waist circumference (TRE and CR) ↓ BMI (TRE and CR) ↑ Insulin sensitivity (TRE)
Schübel et al., 2018 [44]	Overweight/obese non-smokers, *n* = 150 (ICR, *n* = 49/CCR, *n*= 49/CON, *n* = 52); ADF, 49.4 ± 9.0 yrs./CCR, 50.5 ± 8.0 yrs./CON, 50.7 ± 7.1 yrs.; ICR, 49% F/CCR, 49%F/CON, 52%F; BMI: ICR, 32.0 ± 3.8 kg/m^2^/CCR, 31.2 ± 4.0 kg/m^2^/CON, 31.1 ± 3.6 kg/m^2^	ICR, 5:2 diet, (5 d without energy restriction and 2 d with ↓ 75% energy deficit; CCR, daily energy deficit ↓ 20%	12.5 mo	Parallel-group RCT	No significant differences between ICR and CCR regarding various circulating metabolic biomarkers.
Teng et al., 2011 [45]	Healthy men, *n* = 25 (FCR, *n* = 12/CON, *n* = 13); FCR, 59.3 ± 3.4 yrs./CON, 58.3 ± 6.3 yrs.; 0% F; BMI: FCR, 27.0 ± 1.7 kg/m^2^, CON, 25.0 ± 2.9 kg/m^2^	↓ kcal intake to 300–500/day; 2×/week Muslim sunnah fasting (FCR)	3 mo	Parallel-group RCT	↓ Body weight ↓ BMI ↓ Body fat percentage ↓ depression ↑ energy
Teng et al., 2013 [46]	Healthy men, *n* = 56 (FCR, *n* = 28/CON, *n* = 28); FCR, 59.6 ± 5.4 yrs./CON, 59.1 ± 6.2 yrs.; 58.8 ± 5.1 yrs.; 0% F; BMI: FCR, 26.8 ± 1.7 kg/m^2^, CON, 26.7 ± 2.3 kg/m^2^	↓ kcal intake to 300–500/day; 2×/week Muslim sunnah fasting (FCR)	3 mo	Parallel-group RCT	↓ Energy intake (~18%) ↓ Body weight ↓ BMI ↓ Fat percentage ↓ Fat mass ↓ Blood Pressure ↓Total cholesterol ↓ Low-density lipoprotein cholesterol ↓ Ratio of total cholesterol/high-density lipoprotein cholesterol ↓ DNA rejoining cells ↓MDA
Johari et al., 2020 [47]	Individuals w/non-alcoholic fatty liver disease, *n* = 43 (MACR, *n* = 33/CON, *n* = 10); MACR, 45.33 ± 10.77 yrs./CON, 52.60 ± 12.03 yrs.; MACR, 27% F/CON, 10% F; BMI: MACR, 31.60 ± 5.19 kg/m^2^/CON, 28.21 ± 3.32 kg/m^2^	MACR	2 mo	Parallel-group RCT	↓ Body weight ↓ BMI
Cai et al., 2019 [48]	Individuals w/non-alcoholic fatty liver disease, *n* = 264 (ADF, *n* = 90/TRF, *n*= 95/CON, *n* = 79); ADF, 35.5 ± 4.417 yrs./TRF, 33.56 ± 6.23 yrs./CON, 34.54 ± 6.96 yrs.; ADF, 66% F/TRF, 69%F/CON, 52%F; BMI: ADF, 75.32 ± 8.53 kg/m^2^/TRF, 74.98 ± 8.02 kg/m^2^/CON, 72.94 ± 8.00 kg/m^2^	ADF, TRF	3 mo	Parallel-group RCT	↓ Body weight (ADF and TRF) ↓ Fat mass (ADF and TRF) ↓ Total cholesterol (ADF) ↓ Serum triglycerides (ADF and TRF)
Hajek et al., 2021 [50]	Obese individuals, *n* = 284 (5:2SH, *n* = 95/5:2G, *n*= 94/CON, *n* = 95); 5:2SH, 51 ± 13 yrs./5:2G, 47 ± 13 yrs./CON, 47 ± 13 yrs.; 5:2SH, 68% F/5:2G, 67%F/SBA, 64%F; BMI: 5:2SH, 33.4 ± (31.7–37.7) kg/m^2^/5:2G, 34.0 ± (31.7–37.7) kg/m^2^/CON, 34.0 ± (30.7–37.7) kg/m^2^	5:2 Diet	12 mo	Parallel-group RCT	↓ Body weight (5:2SH, 5:2G, and CON)

Note: Study information is presented as reported. ^a^ Groups were excluded in studies that included interventions outside the scope of this review (i.e., exercise). ^b^ Outcome(s) presented as intervention vs. control. Abbreviations: ADF, alternate day fasting; BMI, body mass index; CCR, continuous calorie restriction; CON, control group; CR, calorie restriction; FCR, Fasting Calorie Restriction; FFM, fat-free mass; FMD, fasting-mimicking diet; GLP-1, glucagon-like peptide-1; HDL, high-density lipoprotein; HL, healthy lifestyle guidelines; HOMA-IR, Homeostatic Model Assessment for Insulin Resistance; ICR, intermittent caloric restriction; IF, Intermittent fasting; IER, intermittent energy restriction; IGF-1, Insulin-like growth factor 1; LDL, low-density lipoprotein; MACR, modified alternate-day caloric restriction; RCT, randomized controlled trial; TNF-α, tumor necrosis factor-alpha; TRE, time-restricted eating; TRF, time-restricted feeding. ↑ = increased or improved; ↓ = decreased or reduced.

Notably, the two articles published by this research group employed Muslim sunnah fasting with 300–500 CR over the course of 3 months in a Malaysian population [45,46]. The participants were non-obese (i.e., BMI 23.0–29.9 kg/m^2^) and reported decreases in feelings of depression and increases in energy levels compared with the ad libitum control group [45]. In a larger study, Teng et al. (2013) noted overall cardiometabolic improvement (e.g., blood pressure, lipid profile, and decreased body fat) with significant increases in total rejoining of DNA cells and a decrease in damage of DNA cells and lipid peroxidation.

Of these studies, two assessed various forms of CR (i.e., ADF) in patients with non-alcoholic fatty liver disease, reporting improvements in liver steatosis and fibrosis [47] and body weight [48]. Further, four studies assessed IF and CR regimens for 12 months or longer [38,41,44,50]. Briefly, Trepanowski et al. (2017) looked at ADF vs. CR (75% of energy needs every day) vs. a control group over a 12-month period. The first 6 months were a weight loss period, while the last 6 months were a weight maintenance period. Weight loss and other outcomes such as blood pressure, heart rate, triglycerides, fasting glucose, fasting insulin, insulin resistance, C-reactive protein, or homocysteine concentrations at months 6 or 12 were similar between intervention groups. Though, the dropout rate was highest for the ADF group (38%). Schübel et al. (2018) used the 5:2 model, reducing energy intake two days per week to promote a 20% reduction in energy compared with continuous calorie restriction and a control group (no advice to restrict energy). While both intervention groups lost body weight, the IF group did not exert stronger effects on the adipose tissue transcriptome, circulating biomarkers (of glucose metabolism, lipid metabolism, and inflammation as well as adipokines and steroid hormones), body weight, or VAT and SAT volumes. Moreover, dropout rates are two times lower in the IF group compared with the CR group (i.e., ~8% vs. ~16%). Finally, Lin et al. (2023) compared TRE (eating between noon and 8:00 pm) to CR (25% energy restriction) over a 12-month period. Notably, the TRE group had similar attrition (TRE: 13% vs. CR: 17%) and reduced energy intake and body weight compared with CR (TRE: −4.6 kg vs. CR: −5.4 kg). None of the included studies directly reported on cancer or neurocognitive outcomes.

## 4. Discussion

The current scoping review aimed to explore and understand the existing body of literature on IF and/or CR interventions by investigating their effects on aging-related domains, including cardiometabolic, cancer-specific, and neurocognitive outcomes. The review included 30 articles. Both CR and IF interventions demonstrated significant effects on reduced body weight and fat reduction. IF interventions, including TRE, ADF, and other forms of IF, showed positive impacts on body weight and fat reduction [27,30,35,49,52,53,54,55]. Similarly, studies implementing CR reported consistent reductions in body weight and fat mass [28,29,32,34,37,39,43]. These findings are in line with previous research suggesting the efficacy of both IF and CR interventions in promoting weight loss and managing adiposity [45,46,47,48,50].

Weight loss is a common goal for many individuals, and dietary strategies such as IF and CR have gained popularity due to their potential effectiveness in achieving weight loss. In addition, these dietary strategies may have other potential benefits, such as improving metabolic health and lifespan. It is important to note while not appropriate for all populations (e.g., individuals with active/previous eating disorders, frailty, pregnancy, or advanced age), both IF and CR strategies are generally well-tolerated and demonstrate acceptable safety profiles [13,14]. However, IF may support decreased attrition and long-term sustainability, especially when CR is employed at reductions used by members of CR Society International, a nonprofit organization promoting CR to extend human longevity, as an example (~30% below calorie maintenance requirements) [15]. As an approach to weight loss, CR is far more well-recognized and generally understood to be the equation of consuming fewer calories than one normally would.

As an alternative dietary strategy, IF may offer an approach to improve aging-related outcomes, including cardiometabolic, cancer, and neurocognitive outcomes. The majority of studies populated by the current review focused on cardiometabolic outcomes. Both IF and CR interventions exhibited improvements in blood lipids, glucose metabolism, and insulin sensitivity. IF interventions showed positive effects on blood lipids and glucose metabolism [31,35,49,52,54,55], while CR demonstrated reductions in LDL-cholesterol and total cholesterol/HDL ratio [29]. These findings are promising, indicating the potential benefits of both IF and CR in managing cardiovascular risk factors. Moreover, several studies reported reductions in oxidative stress and inflammation following select IF and CR interventions. Lower levels of CRP were observed in participants engaging in IF or CR [29].

The neurocognitive effects of IF and CR were assessed in a limited number of studies. Both interventions may have potential benefits for neurocognitive health. One study found that CR led to improved recognition memory and altered functional connectivity in specific brain regions [43]. These preliminary findings warrant further investigation into the impact of IF and CR on brain health, cognitive function, and the potential attenuation of neurodegenerative diseases.

Adherence to long-term CR regimens was generally reported to be low, indicating that CR may not be sustainable for most individuals over extended periods. On the other hand, IF interventions demonstrated increased adherence and long-term sustainability, suggesting that IF may be more feasible as a continued and lasting dietary strategy for some populations. Sustainable dietary approaches are crucial for long-term health benefits, and the potential of IF in this regard warrants additional attention. Indeed, as the classic CR study conducted by Ancel Keys in the 1940s reported, depression increases following substantial restriction (i.e., ~45% from baseline maintenance) [58], which may contribute to reduced adherence and long-term sustainability.

Studies combining and/or comparing IF and CR interventions demonstrate promising results on cardiometabolic outcomes, especially in populations with specific health conditions such as NAFLD [47,48]. However, more research is needed to fully understand the feasibility and efficaciousness of combined IF and CR approaches for varied populations and select health outcomes.

This current study provides a comprehensive scoping review of the existing RCT literature on IF and/or CR interventions in adult populations, including cardiometabolic, cancer, and neurocognitive outcomes. However, there are several acknowledged limitations. First, the broad approach adopted in this review may have introduced potential bias in the study selection process and could have influenced the heterogeneity of interventions and outcomes studied. To address these limitations, future research should focus on high-quality RCTs to support the number of studies per outcome and the diversity in methods. Additionally, longer-term studies with larger sample sizes and more diverse populations are necessary to assess the generalizability and sustainability of these dietary strategies. Furthermore, a notable paucity of data in the current scoping review limits the overall findings related to cancer and neurocognitive outcomes. Despite the comprehensive nature of this scoping review, some recent relevant studies may not have been included in the review, and there is a possibility of publication bias affecting the results. Limited data availability resulted in highly heterogeneous data, which is reflective of the current state of the science regarding studies with human participants engaged in IF and/or CR interventions. Moreover, the scarcity of RCTs comparing IF and/or CR interventions to appropriate control groups imposed limitations on the number of applicable studies for the current review. To overcome the noted limitations, future research should focus on conducting additional RCTs that directly compare IF and CR interventions with appropriate control groups. These studies should aim to include larger and more diverse populations, and longer-term follow-up should be incorporated to assess the adherence, sustainability, and long-term effects of these dietary strategies. Furthermore, efforts should be made to reduce heterogeneity among interventions and outcomes through standardized protocols.

## 5. Conclusions

In conclusion, the current scoping review highlights the potential geroprotective effects of IF and CR on cardiometabolic, cancer, and neurocognitive outcomes. Both IF and CR protocols show promise in improving weight loss, blood lipids, glucose metabolism, and insulin sensitivity and reducing oxidative stress and inflammation. While CR has been extensively studied in obesity, IF is a relatively new and understudied dietary strategy that warrants further attention. The findings of this review emphasize the need for more RCTs and robust methodological approaches to understand better the potential mechanisms and long-term effects of IF and/or CR on aging-related outcomes. Ultimately, this research may drive the path for the establishment of evidence-based dietary recommendations and interventions to improve health.

## Figures and Tables

**Table 1:** Review of Terminology.

Caloric restriction:	Overall reduction in calories compared with normative energy intake, generally involving the reduction in energy intake >20% daily without malnutrition.
Intermittent fasting:	Voluntary abstinence of caloric consumption over periods of hours and/or days.
Prolonged nightly fasting:	Daily eating within a timeframe that is in alignment with the biological circadian rhythm (i.e., food/beverage caloric consumption during the active waking hours and abstinence during the nighttime).
Alternate day fasting:	Ingestion of ad libitum energy intake on alternating days coupled with fasting days (i.e., no food/beverage caloric consumption).
Time-restricted eating:	A specific, although flexible, window for daily timing restrictions on eating and fasting.
Periodic fasting: 5:2	May involve fasting for several days (e.g., 2 to 7 days) repeated once per month or heavy restriction of a specific macronutrient (i.e., protein). Routine eating for 5 days followed by 2 (non-consecutive) days of caloric restriction (500–600 calories/day).

## Data Availability

Not applicable.
